# Progression towards smoking cessation: Qualitative analysis of successful, unsuccessful, and never quitters

**DOI:** 10.1080/14659891.2017.1378746

**Published:** 2017-10-05

**Authors:** Ildiko Tombor, Eleni Vangeli, Robert West, Lion Shahab

**Affiliations:** ^a^ Department of Behavioural Science and Health, University College London, London, UK; ^b^ Department of Psychology, London South Bank University, London, UK

**Keywords:** Typology, process of smoking cessation, qualitative

## Abstract

Background: Understanding how people transition between phases of not making a quit attempt to stopping smoking successfully is important in order to optimize interventions. This study aimed to explore differences in attitudes towards smoking and quitting among smokers and ex-smokers. Methods: Adult (age 18 and over) successful (n = 8), unsuccessful (n = 8) and never quitters (n = 7) were recruited through stop-smoking services in England. Semi-structured interviews were conducted and data were analysed using framework analysis. Results: Seven themes (starting to smoke, positive appraisal of smoking, responsibility for past/current smoking, negative effects of smoking, reasons to quit, process of quitting, and identity) were identified in all groups. Sub-group differences were explored and used to derive six typologies with descriptive characteristics: committed smokers, aware smokers, forced attempters, struggling attempters, pragmatic ex-smokers, and committed non-smokers. Using these typologies and the smallest number of differentiating factors between them (awareness of negative effects, motivation to stop and acceptance of responsibility), a parsimonious model of progression towards smoking cessation was developed. Conclusions: Awareness of negative effects, motivation and, crucially, a sense of commitment for taking responsibility to take actions towards behavior change may be important for whether smokers attempt to quit and progress to a successful quit attempt.

## Introduction

Approximately a third of current smokers attempt to quit smoking each year in England (West & Brown, 2012), but even effective behavioral interventions, such as those provided in the UK Stop-Smoking Services (SSS), yield less than one in six successful long-term ex-smokers (Ferguson, Bauld, Chesterman, & Judge, 2005). Many models have been developed to explain the process of behavior change (Michie, West, Campbell, Brown, & Gainforth, 2014a), and various predictors of quit attempts success have been identified (Vangeli, Stapleton, Smit, Borland, & West, 2011). However, it is likely that there exist yet to be uncovered influences, which persist in impeding successful behavior change. Moreover, contextualized information on smoking/quitting behavior of those who engage with intensive behavioral support, including why they attempt to quit and how they quit successfully remains scarce. In-depth information on these processes could provide insights to inform the development and improvement of smoking cessation interventions.

Population studies suggest that motivation to quit is an important predictor of quit attempts (Kotz, Brown, & West, 2013). Barriers and facilitators for quit attempts include concern for immediate/future health and smoking enjoyment (Uppal, Shahab, Britton, & Ratschen, 2013; Vangeli & West, 2008). Importantly, increasing the salience of health concerns (Vangeli & West, 2012), the effects that smoking has on loved ones, the financial burden of smoking (Bethea, Murtagh, & Wallace, 2015; Vangeli & West, 2012) and the internalization of new identity aspects (e.g. mother-to-be) incongruent with ones smoker identity (Bond et al., 2012) can trigger quit attempts. This indicates that identification of processes that enable change in salience of motivation to quit is of key importance for the development of effective health promotion interventions.

Increasing quit attempts and quit success in the population requires a comprehensive strategy (WHO, 2003). It involves a thorough understanding of people’s smoking/quitting behavior, identifying potential intervention targets, utilizing intervention functions that are likely to bring about behavior change, and implementing relevant policies to enable these interventions (Michie, Atkins, & West, 2014b). The Behavior Change Wheel (BCW) (Michie et al., 2014b; Michie, Van Stralen & West, 2011) provides a systematic framework and a comprehensive list of intervention functions and policies to guide this strategy.

Qualitative research can provide important inputs for intervention development (Craig et al., 2008) and theory generation to inform evidence-based practice (Green & Britten, 1998). This study therefore sought to explore how people go from smoking to non-smoking (with a focus on the process mechanisms in cessation) by exploring the attitudes of people who never attempted to quit and those who engaged with the SSS and were either successful or unsuccessful in their quit attempts. The aim was to develop an archetypal description of this process which would be applicable across different tobacco control contexts. The following research questions were addressed in relation to attitudes towards smoking and cessation:

What are the differences and similarities between people who:

1. never attempted to quit and those who made a quit attempt with the help of the SSS
2. quit successfully and who failed in their quit attempt?

## Methods

### Participants

Purposive sampling was used as the aim was to recruit participants on the basis of key characteristics pertinent to the research question (Patton, 2002), and to cover the whole spectrum of smokers. Participants (age 18+) were recruited into one of three groups: 1) ex-smokers who had attempted to quit at the SSS and succeeded at six months following a quit attempt (‘successful quitters’; SQs; n = 8); 2) current smokers who had attempted to quit at the SSS, but failed to achieve six months abstinence (‘unsuccessful quitters’; USQs; n = 8); and 3) current smokers who had never attempted to quit (‘never quitters’; NQs; n = 7). The sample size (n = 23) was considered suitable to achieve saturation, participant diversity and to allow for in-depth analysis (Ritchie, Lewis, & Elam, 2003).

### Procedure

SQs and USQs were recruited through the SSS to ensure the sample included smokers who had engaged with intensive behavioral support to aid cessation. In England, a nationwide network of SSS was established in the late 90s to provide behavioral support with pharmacotherapy free to all smokers at the point of accessing the service (McNeill, Raw, Whybrow, & Bailey, 2005). Everyone (approximately 100 people) who made a quit attempt at the Enfield or Haringey SSS six months prior to the recruitment in 2005 was approached via mail.

Those who returned a registration of interest form by freepost were contacted over the phone and screened against eligibility criteria (age 18+, willing to participate without reimbursement and resident of Enfield or Haringey). The London borough of Enfield is situated in north-east London and borders Haringey to the south. Enfield and Haringey are diverse demographically as approximately half of their population are from ethnic minority groups (www.enfield.gov.uk; www.haringey.gov.uk). Participants’ queries were addressed over the phone, and a date, time and location for the interview (either at the two SSS or the participant’s home) were arranged.

Prior to the interview, consent was recorded and participants completed a background questionnaire. In line with conventions on evaluating long-term effects of smoking cessation treatments, self-reported six months abstinence was chosen to differentiate between SQs and USQs (West, Hajek, Stead, & Stapleton, 2005). The interviews lasted 45–60 minutes and were conducted by LS. NQs were recruited using snowballing technique by asking other participants if they knew any smokers who had never attempted to quit and would be interested in taking part in the study. Identified individuals were asked to contact the research team, and then the same procedure was followed. NQs were given information about available cessation support. Ethical approval was obtained from the Research Ethics Committee of the relevant NHS trusts of the SSS (Reference number: 05/Q0509/28).

### Interview and topic guide

Semi-structured interviews were conducted, and a topic guide (Supplementary Tables S1–3) was used. Three main areas were covered: 1) smoking history, 2) attitudes towards smoking, and 3) thoughts and experiences of quit attempts. The development of the topic guide involved discussions between members of the research team and drew on concepts from a then pre-publication version of the PRIME theory (West, 2006; West & Brown, 2013) available to the authors. This comprehensive theory of motivation encompasses influences on behavior from reflexes and habits through drives to evaluations and plans. Participants were encouraged to speak freely and, if necessary, answers were probed further.

### Questionnaire

To contextualize participants’ accounts, socio-demographic and smoking data were collected (Table 1).

**Table 1.** Participants’ characteristics.

	Total N = 23	Successful quitters N = 8	Unsuccessful quitters N = 8	Never quitters N = 7
**Demographics**				
Age, Mean (SD)	43.9 (14.5)	45.8 (10.0)	50.6 (14.7)	34.1 (15.0)
Female, % (N)	65.2 (15)	62.5 (5)	75.0 (6)	57.1 (4)
White, % (N)	95.7 (22)	100 (8)	87.5 (8)	100 (7)
Higher education, % (N)	69.6 (16)	75.0 (6)	50.0 (4)	85.7 (6)
Paid employment, % (N)	60.9 (14)	62.5 (5)	50.0 (4)	71.4 (5)
Married/living with partner, % (N)	39.1 (9)	37.5 (3)	37.5 (3)	43.9 (3)
**Past/current smoking**				
Cigarettes per day, Mean (SD)	19.7 (7.8)	24.4 (8.2)	17.2 (6.7)	17.1 (7.0)
FTCD (range:0–10), Mean (SD)	4.5 (2.6)	6.1 (2.6)	3.6 (2.5)	3.7 (2.0)

FTCD: Fagerstrom Test of Cigarette Dependence.

### Analysis

Tape-recorded interviews were transcribed *verbatim* and data analysed following the principles of framework analysis (Ritchie & Spencer, 1994). This approach provides a rigorous methodology for analysis, and it has been designed for use in social policy research. Framework analysis takes a ‘subtle realist’ stance regarding data, which assumes that there is an objective reality in the world independent of our understanding of it (ontological position), but this reality can only be accessed through our own perspective of it (epistemological position). The analysis involved: 1) reading and re-reading a subset of transcripts, two from each purposively recruited group randomly selected (familiarisation); 2) identification of concepts and recurrent categories (coding); 3) arranging concepts and categories into sub-themes and key themes (developing the thematic framework); 4) indexing subsequent transcripts using the thematic framework (refining and finalising the thematic framework); 5) creating a matrix, in which every participant is allocated a row and each column denotes a different sub-theme under key themes; and 6) synthesising data from each individual participant within the appropriate part(s) of the thematic framework (charting). The matrix-based approach in framework analysis allows for easy access to synthesized data that can be continually revisited, the ability to look within individual cases across a range of themes, and move between theme- and case-based analysis in order to identify complex layers of patterns in the data and gain in-depth understanding (Gale, Health, Cameron, Rashid, & Redwood, 2013). In the final stage of data synthesis, a ‘top-down’ (theory driven/deductive) data analysis approach was adopted to complement the ‘bottom-up’ (data driven/inductive) text analysis. For the deductive analysis, the BCW framework (Michie et al., 2014b) was used to explore how the proposed model fits within an existing comprehensive framework of intervention strategies; in particular, to identify relevant intervention functions that may be used to bring about behavior change.

Transcripts were coded using Atlas.ti (© Scientific Software Development, Berlin) to identify sub-themes and key themes by LS, with further checks by EV. Following the initial process of developing a thematic framework (EV and LS), charting was carried out by LS. After all data had been charted, descriptive accounts of sub-themes and key themes were produced. This was followed by an in-depth examination of the charts to find salient associations between groups, key themes and demographic characteristics, develop typologies capturing the variation within data, and provide explanatory accounts of the data pattern. Members of the research team discussed each stage of the analysis to minimize the risk of misinterpretation and ensure coherence of the expositions. Deviant case analysis was conducted to scrutinize and refine interpretations.

## Results

Demographic and smoking characteristics (see Table 1) constituted Chart one, which was used for the qualitative sub-group analysis and to develop typologies. Seven key themes, with 22 associated sub-themes, were organized into Charts 2–8 (see Table 2). Not all sub-themes were addressed in equal measure by SQs, USQs and NQs, but each key theme featured in all groups. Key themes are reported first, then sub-group differences, typologies and a simple model of the progression towards cessation.

**Table 2.** Thematic framework.

**Chart 1: Background** Demographic characteristics: age, gender, ethnicity, education, employment and marital status Smoking characteristics: previous quit attempts, current smoking status and tobacco dependence	**Chart 5: Negative effects of smoking** Awareness of smoking-related health risks Minimizing smoking-related health risks Addiction and need for cessation aids acknowledged Addiction and need for cessation aids denied
**Chart 2: Starting to smoke** Extrinsic motivation to initiate smoking Intrinsic motivation to initiate smoking	**Chart 6: Reasons to stop smoking** Intrinsic motivators Health concerns as motivators Extrinsic motivators
**Chart 3: Positive appraisal of smoking** Enjoyment of smoking Smoking to deal with stress Smoking as friend/shield	**Chart 7: Process of quitting** Quitting is hard Quitting is easy Quitting as a challenge
**Chart 4: Responsibility for past/current smoking** Smoking as norm Shifting responsibility Feeling rebellious	**Chart 8: Smoking and identity** Dislike smoking/smokers Transition in lifestyle Dissociation from smoking/regret at starting to smoke Identity/attitudes not changed

### Key themes

#### Starting to smoke

Some participants recounted extrinsic motivations for early experimentation, such as influences of family and friends, alluring images of smokers, peer pressure and trying to fit into a social group. Others emphasized more internally anchored reasons for starting to smoke, such as curiosity.

#### Positive appraisal of smoking

A positive attitude towards smoking was expressed in all groups. Participants reported past/current enjoyment of smoking, and highlighted the perceived utilitarian functions (e.g. for relaxation) that smoking provided for them. For some, cigarettes denoted something like a friend or a shield from the world providing a safe place to withdraw into their own.

“It was a friend, it wasn’t just smoking […] I enjoyed sitting back by myself having a cigarette.” (P10/SQ/female/age 42)

#### Responsibility for past/current smoking

Some participants took responsibility for smoking/quitting; others presented external factors (e.g. smoking as the social norm) as reasons why their quit attempt had failed or never been made, shifting the responsibility to others. They also felt that their rebelliousness prevented them from growing up and taking on adult responsibilities, such as stopping smoking.

“It’s a psychological rebellion in me that I’m pissed off having to work so hard, but it’s the one bloody thing in my life that I can rebel against cos I’m such an organized, orderly, disciplined person so there’s something belligerent inside me that’s saying ‘go on, have a cigarette’.” (P5/USQ/female/age 45)

#### Negative effects of smoking

Participants acknowledged the potential health risks and their vulnerability to smoking-related diseases. However, some refused to accept the health effects of smoking, or tried to minimize these risks and disease susceptibility.

“I’m sure there are some [effects], like my lungs aren’t going to be as happy as they were, but I don’t expect to get any long term health risks.” (P19/NQ/female/age 29)

Despite acknowledging that smoking can be addictive, many participants claimed that they were not addicted and did not need quitting aids. Others acknowledged their addiction to nicotine and the need for cessation support.

“I wouldn’t have been able to do it [stop smoking] without that [patches].” (P16/SQ/female/age 33)

#### Reasons to stop smoking

Intrinsic motivation to stop smoking (e.g. wanting to quit) was repeatedly identified in participants’ accounts. Current/future health concerns, external factors (e.g. moving to a new country) and lifestyle changes (e.g. increasing physical activity) were also mentioned as triggers for quit attempts.

“If I suffered some serious health problems, I would think that is the time to stop.” (P18/NQ/male/age 37)

#### Process of quitting

Many participants perceived cessation as a difficult process, which demands strong personal commitment. Others felt that quitting was easy, and that they enjoyed the challenge being motivated by social comparison with other people who have gone through the same process.

“I did like the fact that I was asked, watching people saying: ‘No I haven’t smoked for a whole week’, and I was thinking: ‘Well, if you can do it, I can do it’.” (P2/UQ/female/age 62)

#### Smoking and identity

Changes in participants’ views and feelings about smoking were identified in all groups. An indication of such change was having negative feelings about oneself as a smoker.

“I have a terrible time of feeling so guilty and cross with myself. I hate it [smoking] now. I don’t want to be a smoker now.” (P9/UQ/female/age 66)

As a consequence of noticing a shift in public opinion towards stigmatisation of smokers, many participants felt ashamed of smoking and some altered their lifestyle (e.g. not smoking in front of others). They also dissociated themselves from their past/current smoking and smoker identity and expressed regret at ever having started.

“If I’d know how hard it was to quit, I never would have started in the first place.” (P12/USQ/female/age 34).

A new non-smoker identity was expressed by some SQs. Others did not feel that they had changed in terms of their identity, even though they quit successfully.

“I’m the same person, but I don’t smoke anymore.” (P3/SQ/male/age 39).

### Progress towards smoking cessation: Sub-group differences

#### NQs versus SQs/USQs

Participants’ accounts varied both between and within groups, but a number of differences were identified between NQs and those who ever attempted to quit. NQs more often provided intrinsic reasons for starting to smoke (e.g. curiosity) rather than extrinsic reasons (e.g. social influence), which were more common among SQs/USQs. Although the utilitarian aspects of smoking were mentioned in all groups, NQs expressed these in emotionally positive or neutral language (e.g. enjoy taking time away from work), whereas people who had attempted to quit, especially USQs, described smoking in more melancholic language.

“There’s an element of loneliness, there’s an element of not being fulfilled. So sometimes my cigarettes fulfil me because I’m bored.” (P2/USQ/female/age 62)

It appeared that NQs wanted to absolve responsibility for taking the first step towards quitting (e.g. external barriers prevented them from stopping).

“If someone came into my life and said ‘look we’re going to do this if you give up smoking’ and if my life wasn’t such a hard slog and there wasn’t so much stress around me, maybe I could do it.” (P8/NQ/female/age 67)

Health risks of smoking and one’s own disease susceptibility and addiction to cigarettes were denied or minimized among NQs. They also had a more negative attitude towards cessation aids than SQs/USQs.

“I would be really weary of taking something that would interfere with my brain chemicals.” (P22/NQ/female/age 26)

Most participants expressed extrinsic factors (e.g. social influence, money) and health concerns as reasons to stop smoking. Intrinsically driven motivators (e.g. wanting to stop for oneself) were not identified among NQs, whereas it was relatively common among SQs/USQs.

“I did it [stop smoking] for myself, I wanted to. No one had a gun to my head.” (P1/SQ/female/age 55)

#### SQs versus USQs

One of the most striking differences between SQs and USQs was that USQs (similar to NQs) made efforts to shift responsibility and blame for their smoking to external factors (e.g. negative life events). These remarks were virtually non-existent in SQs’ accounts. It appeared that the key aspect of the transition towards behavior change was the realization that it is one’s own responsibility to stop smoking, as expressed by SQs who often talked about their commitment to quit for good.

“This time I was determined I was going to do it.” (P11/SQ/female/age 34)

SQs and USQs alike made frequent references to the negative health effects of smoking, including the addictive nature of cigarettes. However, while most USQs (similar to NQs) minimized the negative effects of smoking, SQs acknowledged their own disease susceptibility.

“I always felt it [smoking] was [damaging]. When I used to smoke normal cigarettes I was definitely aware that I would get a cough and feel a bit chesty.” (P16/SQ/female/age 33)

SQs were convinced that they would not have been able to quit unaided, whereas USQs (similar to NQs) questioned the need of using cessation aids (e.g. nicotine replacement therapy).

“The physical side is always the easy side of quitting. My attitude towards smoking is the psychological side. The psychological addiction is the difficult part to crack. (P7/USQ/male/age 37)

SQs and USQs alike reported changes in their attitudes towards smoking (i.e. from positive to negative) and identities (e.g. become someone who does not smoke).

“I want to quit before I’m 35 and never smoke again. I want it to be a pivotal change in my life, where I actually grow up and become an adult. I‘ve been a big kid all my life, a bit of an idiot really.” (P12/USQ/female/age 34)

Although USQs regretted having started smoking and felt ashamed, a shift in identity was more complete among SQs: their former smoker identity was perceived as a separate entity from their current non-smoker/ex-smoker identity.

“Now I can’t believe that I ever smoked.” (P3/SQ/male/age 39)

Nevertheless, this shift in identity was not identified in all SQs’ accounts, as some did not feel that their views or identity changed following cessation.

“I don’t feel a changed person.” (P17/SQ/male/age 55)

#### Synthesis: Derived typology

In order to synthesize the data further, a typology of smokers/ex-smokers was developed. Table 3 reports six identified typologies with related descriptive characteristics. The typologies are necessarily approximate and can therefore not provide a perfect fit for everyone regarding all the characteristics considered therein.

**Table 3.** Identified typologies of smokers and ex-smokers with related characteristics.

‘Committed smokers’ (e.g. participant P23/NQ/male/age 27) never made a quit attempt and considered smoking a core aspect of their life and identity (e.g. “I just feel like that’s [smoking] kind of naturally me.”). They enjoyed smoking, liked being a smoker, and saw no reason to regret having started smoking (e.g. “I love smoking cigarettes […] so I don’t really regret it.”). They denied the health risks of smoking and claimed that they were not addicted to cigarettes. They did not acknowledge their responsibility for not attempting to quit, and felt that their social and physical environment would need to be changed in order for them to quit (“I think what helps a lot is if you are kind of changing your living environment […] and just kind of dump the cigarette with all these changes might be easier.”).

‘Aware smokers’ (e.g. participant P18/NQ/male/age 37) also never attempted to quit and enjoyed smoking, but in contrast to committed smokers, awareness of the health effects and addiction (e.g. “It’s just pure addiction.”) were emphasized. This meant that although they tried to minimize the potential health risks, aware smokers were unhappy about their smoking and regretted having started. Current/future health problems were perceived as potential triggers to quit; however, their social environment was identified as a barrier (e.g. “A lot of people that I work with smoke and that propagates me to decide not to give up smoking.”).

‘Forced attempters’ (e.g. participant P15/USQ/male/age 74) had made a quit attempt in the past. Although they regretted having started smoking, they enjoyed it and felt positive about being a smoker (e.g. “Happy that I smoke […] I do enjoy it because it occupies me and makes me apparently relaxed.”). They were aware of the health consequences of smoking, but they tried to minimize their susceptibility. They shifted responsibility to others for their smoking, and tried to quit primarily for extrinsic reasons without showing any obvious signs of being internally motivated (e.g. “I stopped purely for my wife.”).

‘Struggling attempters’ (e.g. participant P2/USQ/female/age 62) had attempted to quit and regretted having started to smoke. They accepted smoking-related health risks, but attempted to shift the responsibility for continued smoking to external factors (e.g. “I had a car crash and I’m now wearing a soft collar and I had put on weight and that bothered me and I thought to myself right, I’ll have a cigarette instead of food, that was it.”). In contrast to forced attempters, struggling attempters did not enjoy smoking or being a smoker anymore, and were intrinsically motivated to quit (e.g. “[I stopped] because I wanted to rather than had to.”).

‘Pragmatic ex-smokers’ (e.g. participant P17/SQ/male/age 55) quit successfully; however, they used to enjoy smoking and did not regret having started it (e.g. “It actually meant relaxation […] I don’t really regret [starting] as such.”). They took responsibility for their smoking and quitting, but felt that their identity did not change as a result of cessation. They accepted the negative consequences of smoking, and concerns about potential current/future health problems initiated cessation (e.g. “I had a blood pressure check and it was a bit high and I thought well, I know the associations between smoking and cardiovascular problems.”).

‘Committed non-smokers’ (e.g. participant P16/SQ/female/age 33) also quit successfully and used to enjoy smoking, but they regretted ever having started it. They accepted responsibility for their smoking and acknowledged concerns about health problems as triggers for quitting; however, they also had strong intrinsic motivations to stop smoking (e.g. “[I stopped] because I wanted to […] I don’t think you can force someone to give up if they don’t want to give up, it’s their decision.”). They felt that quitting was accompanied not only by external lifestyle changes, but also internal change in terms of their identity (e.g. “I’m a different person. I’ve just grown up I think, that’s the difference.”).

### Synthesis: A model of progression towards smoking cessation

**Figure 1.**
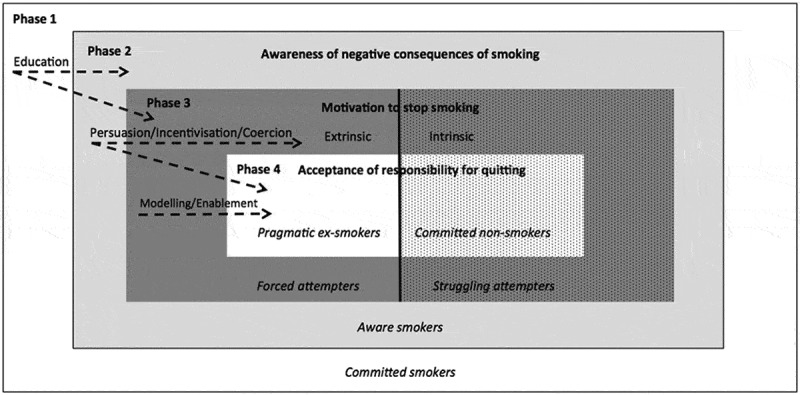
A parsimonious model of the progression towards smoking cessation with potential intervention functions that could influence behavior change. Phases of progression towards cessation are in bold. Typologies are in italics. Solid line represents locus of motivation to stop smoking (solid background = extrinsic; dotted background = intrinsic). Dashed arrows represent intervention functions from the Behavior Change Wheel (BCW)(Michie et al., 2014b) that could influence progression towards smoking cessation. In this model, people may jump phases (e.g. from phase 1 of not attempting to quit smoking to phase 4 of quitting successfully) or move back and forth between phases (e.g. between phases 4 and 3 stopping smoking and then relapsing) being influenced by intrapersonal and environmental factors (e.g., nicotine dependence, availability of support).

In the final step of synthesis, the typologies were used to develop a parsimonious model of the progression towards cessation (Figure 1). The proposed model attempts to coherently classify the various types of smokers/ex-smokers using the smallest number of differentiating factors. As a result, four distinct phases were identified. A phase represents a shorter or longer period of time during the process of cessation and can be described by one of four key activities: 1) not attempting to quit and not being aware of the health consequences of smoking; 2) not attempting to quit, but being aware of the health consequences of smoking; 3) making a quit attempt; 4) stopping smoking. People have to go through the phases in a logical order. A typology determines in which phase an individual is, but a phase does not necessarily determine a typology (except for phases 1 and 2 where only one typology per phase was identified). This model postulates that people rationalize and describe their motivation to change behavior as either linked to external or internal reasons. This is not to say that motivation is external to the person, as it still works through the internal motivational system, but that it can be instigated by external sources. Drawing on the BCW framework (Michie et al., 2014b), the model also suggests potential mechanisms through which interventions might influence behavior change to help smokers progress towards cessation.

Committed smokers deny the negative effects of smoking but, as exemplified by aware smokers, smokers gradually become more cognizant of smoking-related effects even though they may not necessarily admit personal susceptibility. However, without sufficiently strong intrinsic/extrinsic motivation, aware smokers will not attempt to quit. Among those who make a quit attempt, forced attempters are predominantly extrinsically motivated and struggling attempters intrinsically motivated to quit. This model suggests that both types of attempters may fail in part because they have not internalized their personal responsibility for behavior change. Pragmatic ex-smokers and committed non-smokers accept their responsibility to stop smoking and the negative effects of smoking, but they differ in their dominant motivational characteristics, as extrinsic motivation is stronger among pragmatic ex-smokers and intrinsic motivation among committed non-smokers. Therefore, in this model the combination of awareness of the negative effects of smoking, strong extrinsic and/or intrinsic motivation to quit and internalized commitment to behavior change may yield a successful quit attempt.

It is beyond the remit of this analysis to elaborate on the transitions between phases or the influences on the transitions, but it is plausible that smokers may jump phases (e.g. from phase 1 of not attempting to quit to phase 4 of quitting successfully) or move back and forth between phases (e.g. between phases 4 and 3 quitting and relapsing) being influenced by a variety of factors (e.g. nicotine dependence, availability of support). This model argues that smokers’ behavior (both attempting to quit and succeeding in the quit attempt) may be influenced by different intervention functions, including i) education (e.g. increase knowledge and awareness by providing information about the health effects of smoking), ii) persuasion, incentivisation and coercion (e.g. increase motivation to quit by inducing positive/negative feelings and creating expectations of reward/punishment) and iii) enablement and modeling (e.g. increase commitment by providing support and examples for smokers to aspire or imitate).

## Discussion

Our study suggests that awareness of the negative effects of smoking might lay the foundation of the progression towards smoking cessation. Health concerns alone or together with other motivational forces instigated by internal and/or environmental influences could then trigger quit attempts. However, transitioning to long-term cessation might require motivation to be strengthened by a strong personal commitment and change in identity. Six intervention functions (education, persuasion, incentivisation, coercion, enablement and modeling) were proposed to be particularly relevant to target key factors influencing transitions from smoking through quit attempts to quit success. Each of these points is discussed below.

Regarding key differences and similarities between those who have or have not made a past quit attempt, our findings are consistent with previous work (Uppal et al., 2013) showing that unawareness of health concerns is an important barrier to quit attempts. Disregarding negative consequences or derogating one’s own susceptibility to these (McKie, Laurier, Taylor, & Lennox, 2003) can be interpreted within the context of cognitive dissonance theory (Festinger, 1957). It suggests that people can eliminate dissonance either by changing their behavior (e.g. quitting) or altering the associated cognitions (e.g. smoking will not cause health problems for me).

Regarding key differences and similarities between SQs and USQs, this study suggests that one has to go beyond motivation in order to succeed in quitting. Therefore, a subsequent step in the progression towards cessation involves accepting responsibility for one’s behavior. This process can be understood from the vantage point of identity shift theory (Kearney & O’Sullivan, 2003), which proposes that the distress, conflict and dissonance resulting from an unwanted behavior cause a succession of small changes in a person’s attitude and behavior which culminates in identity change. The majority of smokers dislike being a smoker (Tombor, Shahab, Brown, & West, 2013) and this negative feeling can erode one’s self-concept as a smoker, resulting in a new identity as a committed non-smoker. A non-smoker identity may develop over time with the achievement of continued abstinence (Vangeli & West, 2012). Making this mental transition and establishing a non-smoker identity appears to be an important predictor of smoking abstinence in the medium term (Tombor, Shahab, Brown, Notley, & West, 2015a). A smoker identity can also be an important aspect of one’s social identity thus being part of a group of smokers may outweigh the risks of smoking and undermine quit attempts (Oyserman, 2009; Tajfel & Turner, 1986; Tombor et al., 2015b).

This study further elucidates an alternative pathway towards cessation, one which is not closely tied to identity change, but rather, as exemplified by pragmatic ex-smokers, to a realization of the necessity of quitting and the futility of shifting responsibility to others. This behavior change thus appears less connected with internal changes than it is with extrinsic motivators and would, at least in part, explain the effectiveness of policy changes such as increasing taxation on cigarettes or introducing smoking bans (WHO, 2003).

One of the study limitations is that participants were recruited from two geographically and demographically similar SSS using snowballing, resulting in a homogenous sample which is not representative of the UK adult smoker population. While findings may therefore not extrapolate to the broader context, this research still provides an important source for theoretical guidance to inform the development of evidence-based smoking cessation interventions. Another limitation is that data were collected in 2005. However, as we sought to develop an invariant archetypal description of the process of cessation, the proposed typologies and model are still relevant despite recent changes in the smoking cessation landscape (e.g. availability of e-cigarettes).

Regarding practical implications, our model suggests that smokers first need to be made aware of the negative consequences of smoking, consistent with other work in this area (Prochaska, Norcross, & DiClemente, 1994). However, in agreement with PRIME theory (West, 2006; West & Brown, 2013), beliefs about what should be done (e.g. stop smoking given awareness of the negative consequences) will not bring about behavior change unless they generate strong enough motivation. Therefore, individual- and population-level interventions need to provide information about smoking-related health effects and increase the salience of extrinsically and intrinsically anchored motivational forces to influence people’s progression towards cessation by making information personally relevant (see self-determination theory (Ryan & Deci, 2000)). This highlights the importance of mass media campaigns, such as the UK Stoptober campaign, that was framed around a positive message of stopping smoking for 28 days to reach those who are not currently considering making a quit attempt (Brown et al., 2014), While it is difficult to deduce which factors are necessary and sufficient for long-term abstinence, smokers’ commitment to take responsibility for their quitting behavior signals a critical shift towards cessation. This implies that intervention approaches that involve restructuring cognitive patterns, provide role models for cessation, establish a new non-smoker identity and promote commitment to personal rules in which smoking is not an option (e.g. ‘not-a-puff’ rule) are important (Shahab & Kenyon, 2013).

In conclusion, this study identified six distinct smoking-related typologies which exist in different phases of the quitting process. We suggest that people’s knowledge about the negative effects of smoking, their motivation to a quit and personal commitment to behavior change represent important targets for future interventions and related policies to help people transition from phases of not attempting to quit to eventually succeeding in their quit attempts.

## Supplementary Material

Supplemental_Tables_Progression_towards_smoking_cessation_suppl_additional.docx
