# Supplementary material for: Progression towards smoking cessation: Qualitative analysis of successful, unsuccessful, and never quitters
Source: J Subst Use. 2017 Oct 5;23(2):214–22. doi: 10.1080/14659891.2017.1378746 (PMC5827703; doi:10.1080/14659891.2017.1378746)
Supplement: Supplemental_Tables_Progression_towards_smoking_cessation_suppl_additional.docx [file IJSU_A_1378746_SM3047.docx]

Table S1: Topic guide used in semi-structured interviews with ex-smokers who quit successfully (‘successful quitters’)

| **Main topics** | **Example questions** | **Probes** |
| --- | --- | --- |
| 1. Past smoking history | ‘Can you tell me a bit about yourself and how you came to start smoking?’ | - For how long have you smoked? - What initiated smoking (because of friends/family)? - Do you remember the situation in which you had your first cigarette ever? - Do you remember what your intention was regarding smoking (to try it, to become a smoker etc.)? - Do you remember who else was there, - Do you remember where you got the cigarette from? - Do you remember why you started? What was your motivation (curiosity, to be sociable, to be like others etc.)? - How long did it take before you went on to daily smoking? - Aware of health effects at the time? Did you expect to stop before the damage was done? |
| 2. Attitude towards smoking | ‘How did you feel about your smoking then and now?’ | - Smoking because of boredom/to socialise/to keep weight down/for pleasure/to cope? - Why do you think people smoke? - Does your attitude towards smoking change/stay the same from day to day? - Do you still get urges to smoke sometimes? (If so, what do you do?) - Are you happy / unhappy you have quit? - If you were to compare smoking cravings, what did they feel like? Similar to hunger, thirst? - How often, if ever, did you find yourself lighting up without even thinking about it? - Do you accept that smoking is bad for you/your health and your environment? - Did/do you think smoking is going to kill you? - Do you regret having started? - If applicable: How would you feel if your children started smoking? |
| 3. Thoughts on quit attempts | ‘Could you tell me about your quit attempt? What do you think helped you succeed?’ | - Was it easy? Did you feel cravings or similar sensations? If yes, what happened to them? - Did you feel confident in your ability to quit before? - Would you say you stopped because you really wanted to or because you felt you had to? - Why did you quit smoking? Was there a key moment for decision that made you think ‘I really have to quit’/that triggered the quit attempt? - Physical or Mental health issues? Social pressure? - Did you formulate a plan to quit in advance? - Did you make or notice any changes in your life during your quit attempt? - Feel like a different person now? - How would you describe yourself now in terms of smoking? |
| **Ending the interview**   - Anything else participants would like to say? Any important issues not raised? - Any other questions regarding research? - Request if they know any smokers who haven’t attempted quitting who would be interested to participate in the study - Provision of researcher contact information, should anything else come up - Thanks | | |

Table S2: Topic guide used in semi-structured interviews with current smokers who had attempted to quit smoking but failed to achieve abstinence (‘unsuccessful quitters’)

| **Main topics** | **Example questions** | **Probes** |
| --- | --- | --- |
| 1. Past smoking history | ‘Can you tell me a bit about yourself and how you came to start smoking?’ | - For how long have you smoked? - What initiated smoking (because of friends/family)? - Do you remember the situation in which you had your first cigarette ever? - Do you remember what your intention was regarding smoking (to try it, to become a smoker etc.)? - Do you remember who else was there, - Do you remember where you got the cigarette from? - Do you remember why you started? What was your motivation (curiosity, to be sociable, to be like others etc.)? - How long did it take before you went on to daily smoking? - Aware of health effects at the time? Did you expect to stop before the damage was done? |
| 2. Attitude towards smoking | ‘What do you think about your smoking/ smoking in general?’ | - Smoking because of boredom/to socialise/to keep weight down/for pleasure/to cope? - Why do you think people smoke? - What would you miss most if you stopped smoking? - Are you happy/unhappy you smoke? - Does your attitude towards smoking change/stay the same from day to day? - How often, if ever, do you find yourself lighting up without even thinking about it? - Do you accept that smoking is bad for you/your health and your environment? - Do you ever think about the health consequences of smoking & if so, how often? - Do you think smoking is going to kill you? - Do you regret having started? - If applicable: How would you feel if your children started smoking? |
| 3. Thoughts on quit attempts | ‘Could you tell me about your quit attempt? Why do you think it failed?’ | - Was it easy/hard at first? Did you feel cravings or similar sensations? If yes, what happened to them? - Did you feel confident in your ability to quit before? - Would you say you attempted to stop because you really wanted to or because you felt you had to? - Why did you attempt to quit smoking? Was there a key moment for decision that made you think ‘I really have to quit’/that triggered the quit attempt? - Physical or Mental health issues? Social pressure? - Did you formulate a plan to quit in advance? - Did you make or notice any other changes in your life during your quit attempt? - When did you lapse and why did you lapse? - What did you think when you lapsed? - Did you decide to give up on your quit plans or did you try to hang in there? - Would you like to quit? How often do you think about it? - What would it take for you to be absolutely confident not smoker ever again? - How would you describe yourself now in terms of your attitude to smoking? |
| **Ending the interview**   - Anything else participants would like to say? Any important issues not raised? - Any other questions regarding research? - Request if they know any smokers who haven’t attempted quitting who would be interested to participate in the study - Provision of researcher contact information, should anything else come up - Thanks | | |

Table S3: Topic guide used in semi-structured interviews with current smokers who had never attempted to stop smoking (‘never quitters’)

| **Main topics** | **Example questions** | **Probes** |
| --- | --- | --- |
| 1. Past smoking history | ‘Can you tell me a bit about yourself and how you came to start smoking?’ | - For how long have you smoked? - What initiated smoking (because of friends/family)? - Do you remember the situation in which you had your first cigarette ever? - Do you remember what your intention was regarding smoking (to try it, to become a smoker etc.)? - Do you remember who else was there, - Do you remember where you got the cigarette from? - Do you remember why you started? What was your motivation (curiosity, to be sociable, to be like others etc.)? - How long did it take before you went on to daily smoking? - Aware of health effects at the time? Did you expect to stop before the damage was done? |
| 2. Attitude towards smoking | ‘What do you think about your smoking/ smoking in general?’ | - Smoking because of boredom/to socialise/to keep weight down/for pleasure/to cope? - Why do you think people smoke? - What would you miss most if you stopped smoking? - Are you happy/unhappy you smoke? - Does your attitude towards smoking change/stay the same from day to day? - How often, if ever, do you find yourself lighting up without even thinking about it? - Do you accept that smoking is bad for you/your health and your environment? - Do you ever think about the health consequences of smoking & if so, how often? - Do you think smoking is going to kill you? - Do you regret having started? - If applicable: How would you feel if your children started smoking? |
| 3. Thoughts on quit attempts | ‘Why do you think you have never seriously attempted to stop smoking?’ | - Would you like to quit? How often do you think about it? (If so, why have you not attempted to quit?) - Is there pressure on you not to quit? - Do you feel confident in your ability to quit? - Are you afraid that you would not be able to quit? - What would it take for you to be absolutely confident not smoker ever again? - Have you ever formulated a plan to quit/thought about quitting? - How would you describe yourself now in terms of your attitude to smoking? |
| **Ending the interview**   - Anything else participants would like to say? Any important issues not raised? - Any other questions regarding research? - Request if they know any smokers who haven’t attempted quitting who would be interested to participate in the study - Provision of researcher contact information, should anything else come up - Thanks | | |
